# Machine learning models for predicting post-cystectomy recurrence and survival in bladder cancer patients

**DOI:** 10.1371/journal.pone.0210976

**Published:** 2019-02-20

**Authors:** Zaki Hasnain, Jeremy Mason, Karanvir Gill, Gus Miranda, Inderbir S. Gill, Peter Kuhn, Paul K. Newton

**Affiliations:** 1 Department of Aerospace and Mechanical Engineering, Viterbi School of Engineering, University of Southern California, University Park Campus, Los Angeles, CA, United States of America; 2 Department of Biological Sciences, Dornsife College of Letters, Arts, and Sciences, University of Southern California, University Park Campus, Los Angeles, CA, United States of America; 3 USC Institute of Urology, Keck School of Medicine, University of Southern California, Health Sciences Campus, Los Angeles, CA, United States of America; 4 Department of Biomedical Engineering, Viterbi School of Engineering, University of Southern California, University Park Campus, Los Angeles, CA, United States of America; 5 Norris Comprehensive Cancer Center, Keck School of Medicine, University of Southern California, Health Sciences Campus, Los Angeles, CA, United States of America; 6 Department of Mathematics, University of Southern California, University Park Campus, Los Angeles, CA, United States of America; National Cancer Center, JAPAN

## Abstract

Currently in patients with bladder cancer, various clinical evaluations (imaging, operative findings at transurethral resection and radical cystectomy, pathology) are collectively used to determine disease status and prognosis, and recommend neoadjuvant, definitive and adjuvant treatments. We analyze the predictive power of these measurements in forecasting two key long-term outcomes following radical cystectomy, i.e., cancer recurrence and survival. Information theory and machine learning algorithms are employed to create predictive models using a large prospective, continuously collected, temporally resolved, primary bladder cancer dataset comprised of 3503 patients (1971-2016). Patient recurrence and survival one, three, and five years after cystectomy can be predicted with greater than 70% sensitivity and specificity. Such predictions may inform patient monitoring schedules and post-cystectomy treatments. The machine learning models provide a benchmark for predicting oncologic outcomes in patients undergoing radical cystectomy and highlight opportunities for improving care using optimal preoperative and operative data collection.

## Introduction

Bladder cancer (BCa) is the 6th most common cancer in the U.S, with an estimated 79,030 new cases and 16,870 deaths in 2017 [1] and has a 5-year relative survival rate of 79% [2]. BCa staging is based on the TNM system (tumor, nodes, metastasis). In BCa, the “T” stage is dictated by how deep the tumor invades into the various layers of the bladder wall. Ta represents a noninvasive papillary tumor, while T1, T2, T3 and T4 stages represent more aggressive cancers invading the sub-epithelial tissue, muscle, peri-vesical fat and adjacent organs, respectively. Radical surgery is the primary treatment method for invasive cancer and may be augmented with other forms of therapy such as chemotherapy to treat more advanced and aggressive cancers [3]. Radical cystectomy, the recommended method for treating invasive BCa [4], is surgical removal of the bladder, regional lymph nodes and adjacent organs (prostate, uterus, etc.) which may contain cancer. Technical precision of this surgical operation can dictate long-term oncologic outcomes, for instance, post-cystectomy survival is higher when negative surgical margins are obtained and more than ten pelvic lymph nodes removed during radical cystectomy [5]. Conversely, cancer recurrence rates are higher with positive margins and removal of less than ten nodes.5 Furthermore, patients with organ-confined disease are less likely to relapse beyond 5 years, and unlikely beyond 10 years after cystectomy, even without adjuvant treatment [6].

These trends are derived from focused studies with disparate cohorts. The large size of the current dataset offers a chance to confirm and refine these relations. Beyond knowledge discovery, larger and electronically managed medical databases lend to predictive tool development. Consequently, machine learning techniques have been applied extensively on clinical, epidemiological, and molecular data to predict prognosis and outcome in various cancers. Cruz and Wishart [7], and more recently, Kourou et al. [8] offer a review of some of these studies which predict of susceptibility, recurrence and survival, where the merit of techniques and the quality of the data are quantified by prediction accuracies. In BCa, the most relevant existing study used a multi-institution dataset of 9000 patients, including 980 data points from the present dataset, to construct a nomogram for predicting 5-year recurrence which achieved a concordance index of 0.75 [9].

The present work focuses on: (i) using preoperative and operative BCa data to uncover patterns of long term outcomes and (ii) assessing the predictive power of BCa-specific factors in elucidating overall survival (OS) and recurrence. We employ the information theory concept of mutual information (MI) to uncover correlated parameters. We then stratify the set of predictors by correlation with 5-year binary recurrence and binary OS to quantify their relative importance. The prognostic power of these variables is assessed by developing a machine-learning classification pipeline to predict recurrence and survival after radical cystectomy, urinary diversion and extended lymphadenectomy, the standard-of-care for high-risk, muscle-invasive BCa. The models presented deliver a quantitative method for stratifying patients into higher resolution risk groups than is possible with current methods.

## Methods

### Data summary

The original dataset (details in Table A in S1 File) comprised of 3503 patients is pruned to 3499 (mean age 67.8 years) patients by removing 4 cases with missing survival data. All patients underwent radical cystectomy at the USC Institute of Urology from 1971 to 2016. Statistical results based on this dataset up to 1997 were published by Stein et al. in 2001 [10] on a subset of 1054 urothelial carcinoma patients. Presently, this is one of the largest known single-institute datasets of BCa cystectomy patients in terms of sample size and the 45-year timespan over which the data were prospectively and continuously collected with University of Southern California Institutional Review Board (IRB) approval. Consequently, the evolution of preoperative and operative assessments is also explored. In addition to information pertinent to BCa, comorbidity data were also collected to study the effect of preexisting diseases on progression of BCa. Remainder of the data is comprised of demographics, clinical diagnostic information prior to cystectomy, tumor markers prior to cystectomy, and pathologic and surgical data at time of cystectomy including adjuvant therapy treatment information. In the context of machine learning, these preoperative and operative measurements are called predictors, and the target variables are binary indicator variables for recurrence and survival after a given number of years post-cystectomy.

### Statistics and information theory

We perform survival analysis using the Kaplan-Meier estimator to differentiate OS by various predictors. However, to develop an understanding of system-wide patterns between all the predictors, recurrence, and OS, a network approach is more suitable. Relevance, or correlation networks [11–14] can be created using a similarity measure. Therefore, we create a mutual information (MI) network and subsequently a Euclidean distance based complete-linkage agglomerative hierarchical clustering of the most closely associated variables. Here, we use normalized MI which ranges from 0 to 1 for entirely unrelated to maximally related pairs of variables. Larger values of MI correspond to higher dependence between two variables. We normalize MI by the maximum entropy of the two variables being compared [15], and normalized MI will be abbreviated as MI. The set of all pairwise MI relations make up the adjacency matrix of the MI network, which is visualized as a clustered heat-map. For this analysis we limit the dataset to 2618 patients whose recurrence and survival data is known for five years post-cystectomy.

The predictors are ranked by their association with the two long-term 5-year binary outcomes, recurrence and OS, using the chi-squared test of independence which measures the association between two categorical variables. Age, and other continuous variables are discretized to perform the chi-squared test. The composite assessment identifies higher fidelity variables and encapsulates the clinical relevance of the measurements. A composite predictor ranking,

$$\begin{array}{c} rank_{i}=\sqrt{{\left({\bar{\chi }}_{Rec,i}^{2}\right)}^{2}+{\left({\bar{\chi }}_{OS,i}^{2}\right)}^{2}} \end{array}$$ (1)

based on the chi-squared values for recurrence ($\chi_{Rec}^{2}$),

$$\begin{array}{cc} {\bar{\chi }}_{Rec,i}^{2} & =\frac{\chi_{Rec,i}^{2}}{\sigma_{\chi^{2},\text{Rec}}} \end{array}$$ (2)

and the OS chi-squared values ($\chi_{OS}^{2}$),

$$\begin{array}{cc} {\bar{\chi }}_{OS,i}^{2} & =\frac{\chi_{OS,i}^{2}}{\sigma_{\chi^{2},\text{OS}}} \end{array}$$ (3)

is used to identify predictor importance. The chi-squared values for both outcomes are normalized by their respective standard deviations (Eqs 1–3) to weigh the effect of both outcomes equally. Appendix C in S1 File describes the datasets used for this analysis.

### Machine learning approach

The performance of multivariate predictive models is compared to univariate logistic regression models. To create the multivariate models, a series of base predictive models are employed, subsequently mixture-of-experts and stacking based ensemble models are trained using these base models. The base models consist of: support vector machines (SVM), bagged SVM, K-nearest neighbor (KNN), adaptive boosted trees (AdaBoost), random forest (RF), and gradient boosted trees (GBT). The mixture-of-experts models are based on hard-voting among the base models, whereas the stacking ensemble models perform dimensionality reduction of the base model predictions before employing a second logistic regression or support vector machine (SVM) model.

For each prediction task, a different triplet of models forms the final meta-classifier, which is constructed by combining one each of the best base, mixture-of-experts, and stacking classifiers using hard-voting. This method of combining various models is achieves the highest performance metrics.

Patients who leave the study before the target year of the survival models are removed from the dataset, resulting in n = 3201, 3066, and 2780 patients for the 1-, 3-, and 5-year survival datasets respectively. However, only patients who have no recurrences and leave the study before the target year of the recurrence models are removed from the corresponding models’ datasets; resulting in n = 3071, 2955, and 2695 patients in the 1-, 3-, and 5-year recurrence datasets respectively. To avoid class imbalance while training, the subset of patients who recur are randomly oversampled to yield an equal count of recurring and non-recurring patients in the training sets. Similarly, for the survival classifiers, the fraction of surviving and non-surviving patients is balanced by random oversampling.

The procedure for feature selection consists of two steps: removal of irrelevant predictors and removal of redundant predictors. To remove redundant predictors, the hierarchical clustering of the 73 predictors is used to define 60 predictor clusters, and the predictor with the highest MI with the target variable is selected from each cluster. Subsequently, to remove irrelevant predictors from the dataset, predictors with low MI with the target variables are excluded from the dataset (MI<0.006 for predicting recurrence, MI<0.003 for predicting survival). These two feature selection steps yield a set of 52, 54, and 51 predictors for the 1-, 3-, and 5- year recurrence models respectively, and 42, 45, and 45 predictors for predicting 1-, 3-, and 5-year survival respectively.

Final performance scores are found using nested cross-validation with ten outer folds and five inner folds in which the SVM, RF, GBT, and AdaBoost hyper-parameters are tuned. The Scikit-Learn platform [16] is used to implement the models.

## Results

### Survival statistics

**Fig 1:**
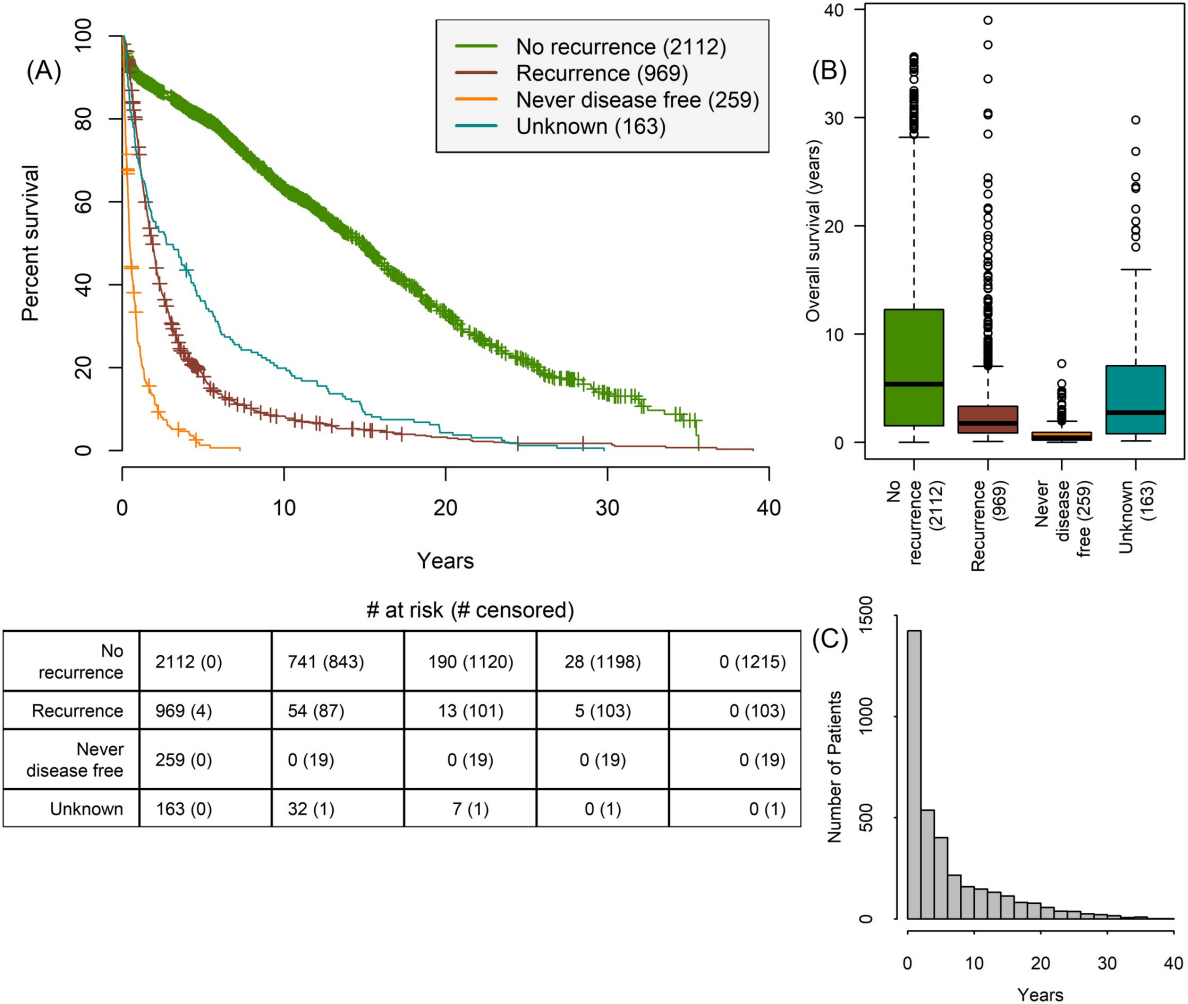
Relationship between disease status and survival. (A) Kaplan-Meier survival for patients who never recurred (green, n = 2112, 60.3%) have the highest mean OS 7.93 years (SD = 7.69), patients who were initially disease free but then had recurrence (red, n = 969, 27.7%) have mean OS 3.00 years (SD = 4.14), patients who were never free (orange, n = 259, 7.4%) of the disease have the lowest mean OS 0.81 years (SD = 1.03), and patients whose post-cystectomy progression is unknown (teal, n = 163, 4.7%). (B) Boxplot of OS by disease progression of patients. (C) Histogram of OS in cohort show a dramatic reduction in survival rates up to five years post-cystectomy.

**Fig 2:**
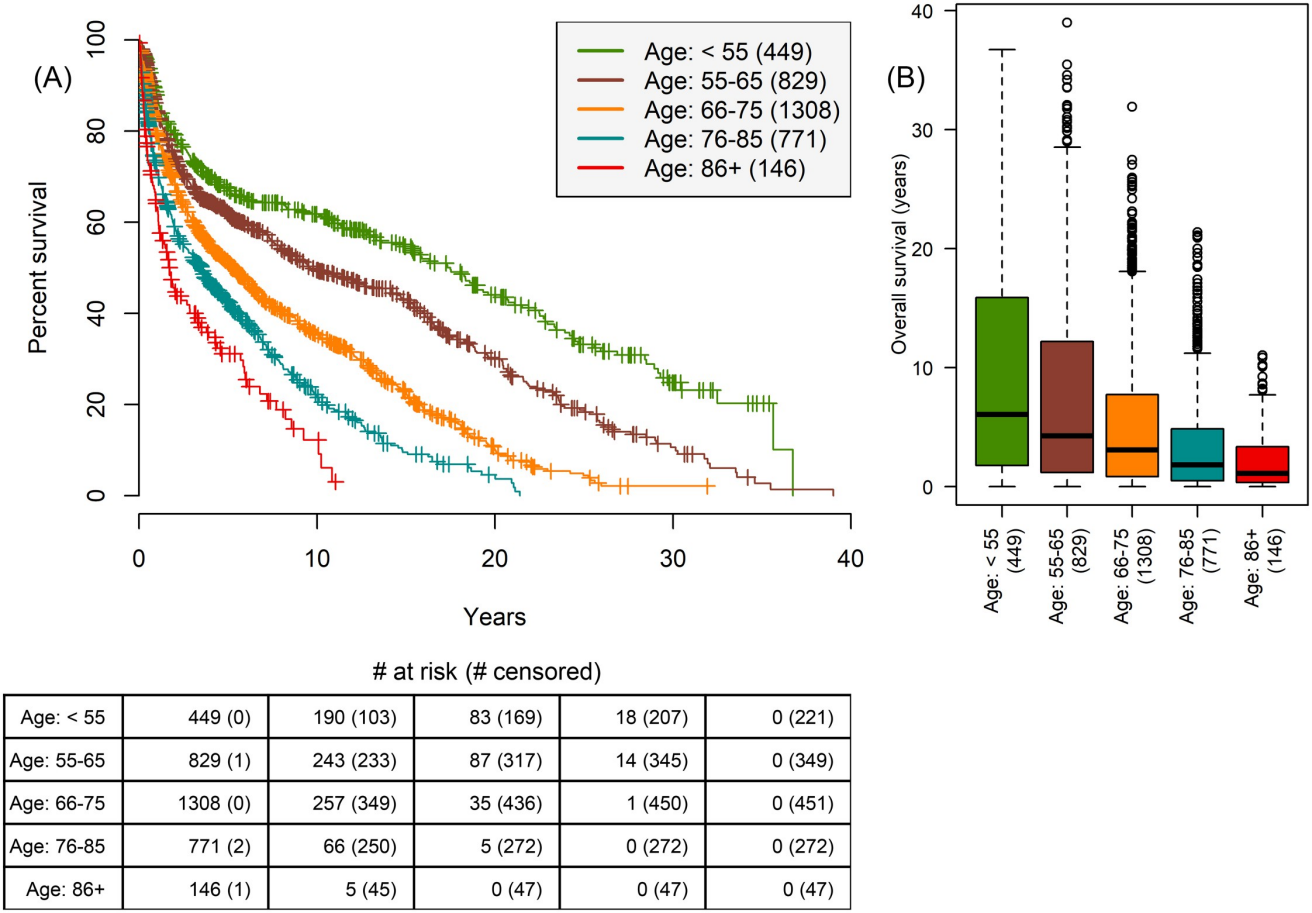
Relationship between age and survival. Patients are stratified into five age groups, according to the Surveillance, Epidemiology, and End Results (SEER) program age standards for survival [17]. (A) Boxplot of OS for patients by age group. Age < 55 (n = 449 patients, 12.8%), 55 ≤ age < 65 (n = 829 patients, 23.7%), 65 ≤ age < 75 (n = 1308 patients, 37.3%), 75 ≤ age < 85 (n = 771 patients, 22.0%), 85 ≤ age (n = 146 patients, 4.2%). (B) Kaplan-Meier survival by disease progression shows disease burden greatly diminishing five years after surgery.

3503 patients’ OS is outlined in Fig 1 and patients with unknown recurrence status are excluded from analysis pertaining to recurrence in the rest of the study. There is an exponential decay in survival by age groups in the five-year period post-cystectomy, which suggests the burden of BCa diminishes significantly within five years for patients undergoing radical cystectomy (Fig 2). Consequently, our prediction tasks focus on 1-, 3-, and 5-year survival and recurrence.

**Fig 3:**
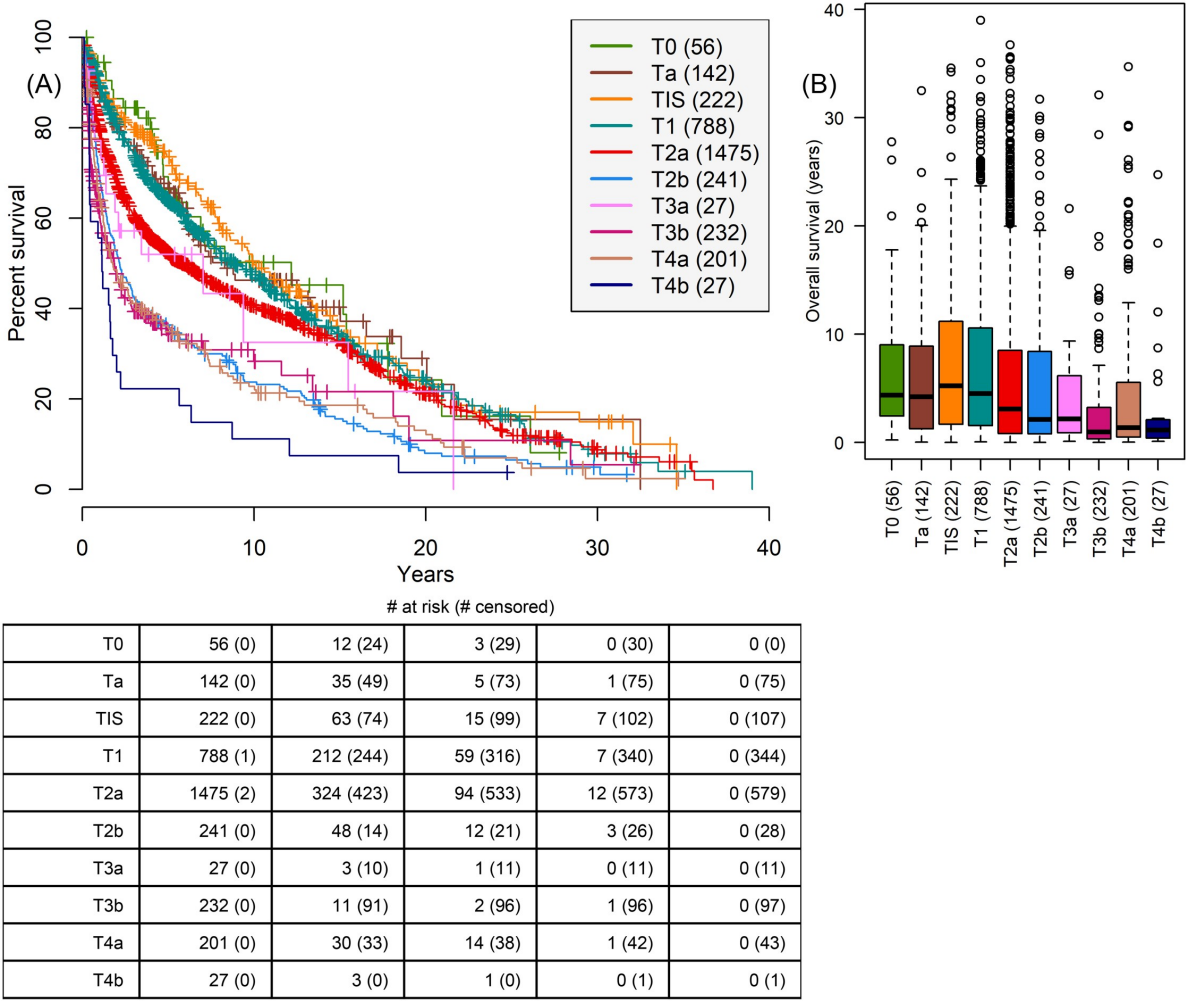
Relationship between survival and clinical T stage assigned prior to cystectomy. Based on a combination of imaging and transurethral resection. (A) Kaplan-Meier survival shows some degradation with tumor staging, but many stages overlap significantly. T3a and T2b patients in the (B) boxplot of OS by T stage are not differentiated. P-values in Table H of S1 File.

**Fig 4:**
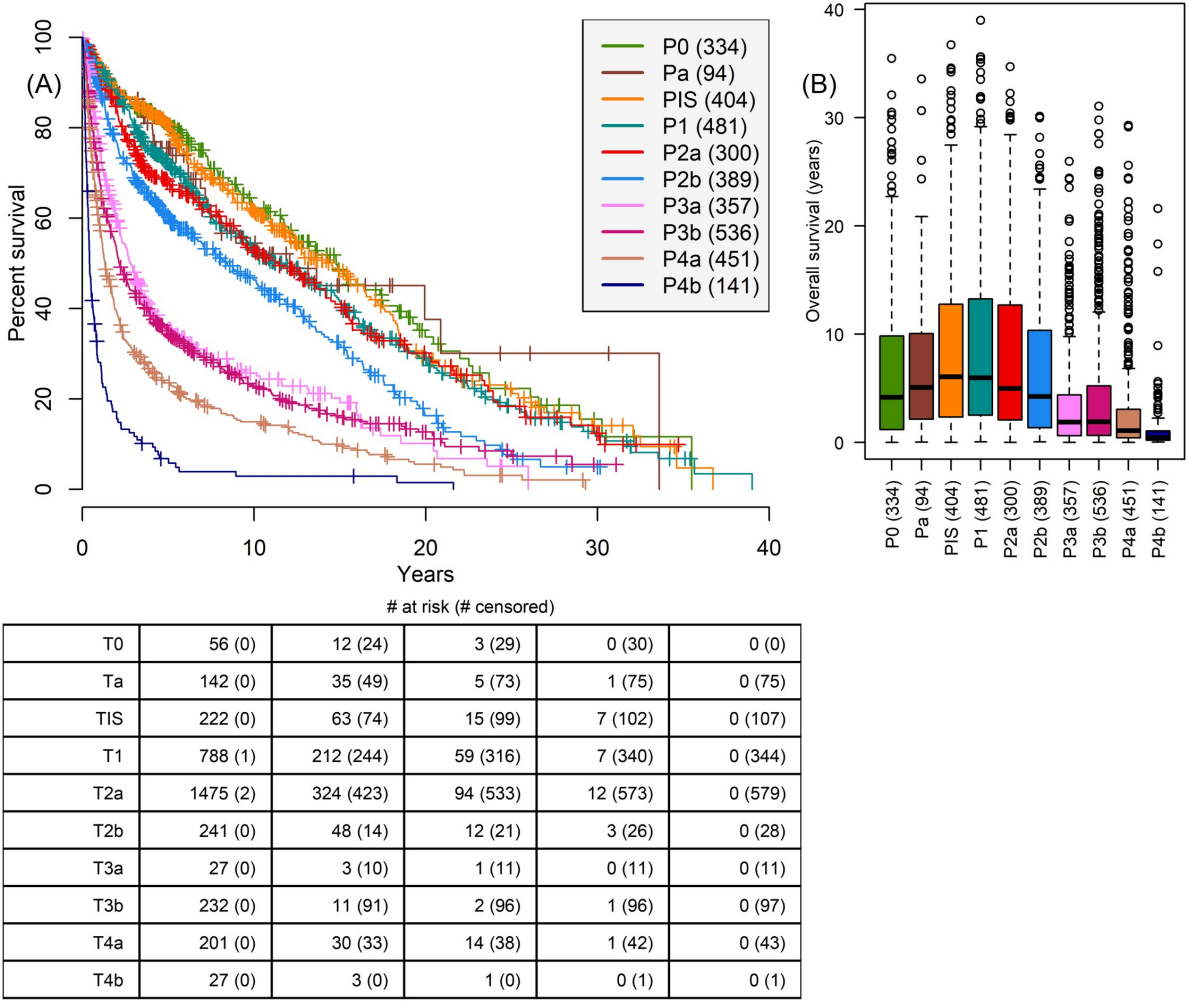
Relationship between survival and pT stage (TNM 5th edition) assigned at time of cystectomy. (A) Kaplan-Meier survival shows a steadier degradation with tumor staging than clinical staging. (B) Boxplot of OS by pT stage at time of cystectomy clearly differentiates P3a and P3b patients from stage two patients. P-values in Table I in S1 File.

Comparing survival for clinical staging prior to surgery (Fig 3) and pathologic staging (pT staging: TNM 5th edition staging) at time of cystectomy (Fig 4) reveals the higher fidelity of pathologic staging. Clinical staging fails to separate staging as clearly as pathologic staging, for example clinical staging does not separate T2b and T3a patients as clearly as pathologic staging P2b and P3a patients.

During the study (1971-2016) there is a 24% (811/3417) agreement between the two staging measures, with clinical staging over-estimating pathologic stage by 25% (865/3417 patients). Since 2010, there is a surge in stage over-estimation, with a corresponding decrease in under-estimation; however, overall concordance between the two staging measures has remained relatively constant over the decades studied (Fig A in S1 File). A graver consequence of the inferior resolution of clinical staging is that it underestimated pathologic stage in 51% of patients (1741/3417).

Patients with organ-confined (OC), extra-vesical (EV) and node-positive (N+) BCa had 5-year survival rates of 0.750 (95% CI [0.729, 0.772]), 0.413 (95% CI [0.377, 0.452]), and 0.213 (95% CI [0.186, 0.243]), respectively (p<0.001) (Fig B in S1 File). Patients with and without lympho-vascular invasion had 5-year survival rate of 0.299 (95% CI [0.271, 0.330]) and 0.637 (95% CI [0.617, 0.658]), respectively (p<0.001) (Fig C in S1 File).

Patients with a negative soft tissue surgical margin had 5-year survival of 0.572 (95% CI [0.553, 0.591]) compared to 0.358 (95% CI [0.285, 0.448]) for patients with positive ureteral/urethral margins and 0.063 (95% CI [0.034, 0.117]) for patients with soft-tissue margins (p < 0.001) (Fig D in S1 File).

Patients with carcinoma in situ had no discernable difference in OS as well as 5-year probability of survival compared to other patients.

### Correlations among predictors

**Fig 5:**
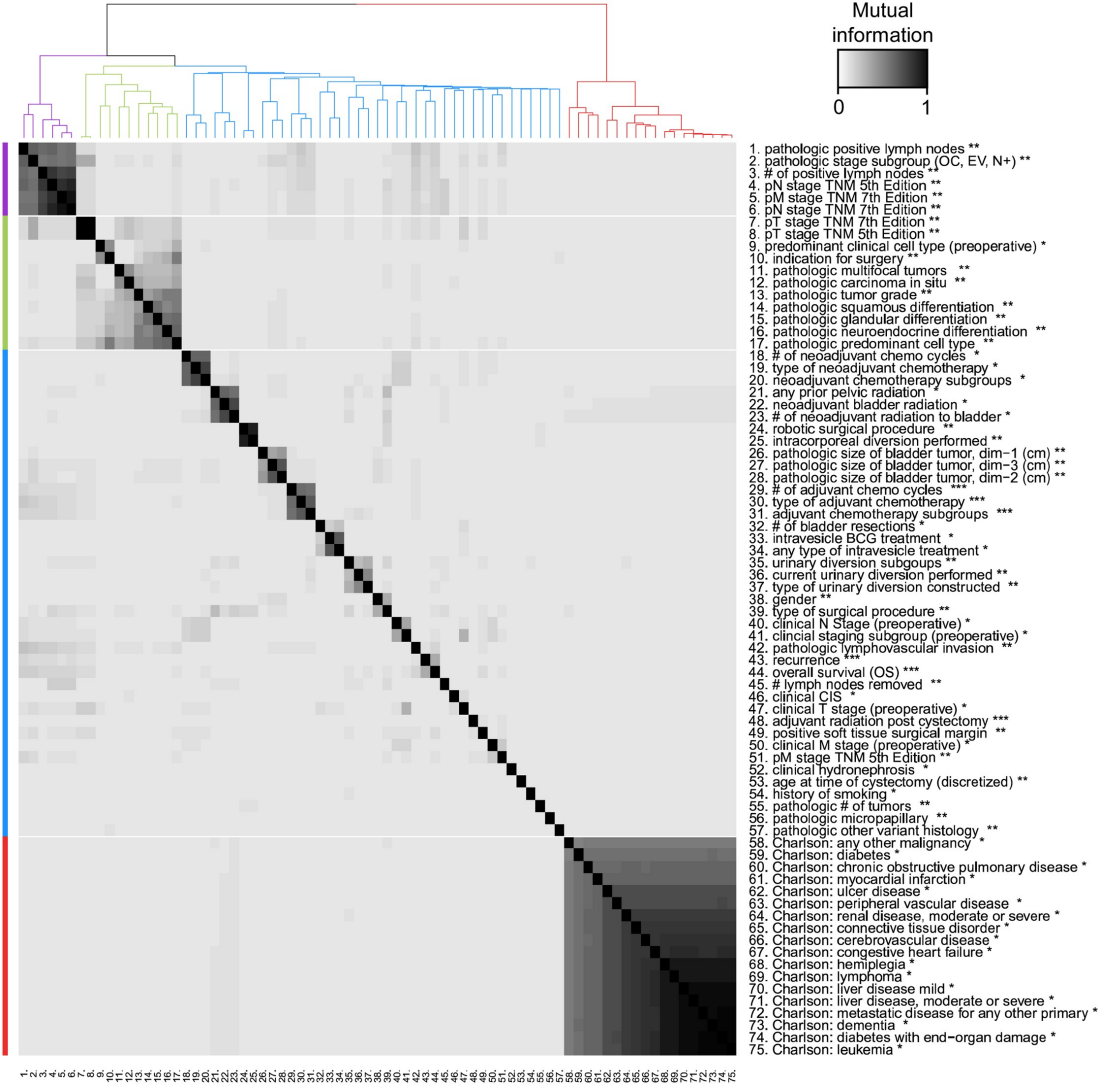
MI between the set of predictors, binary recurrence, and binary OS five years post-cystectomy. Predictors are clustered into four groups using a hierarchical clustering algorithm to discover associated predictor groups. BCa predictors and long-term outcomes are contained in the purple (anatomic staging), green (histologic staging), and blue (treatment and OS and recurrence) clusters, whereas the comorbidity factors comprise a solitary (red) cluster. Correlations within the purple and red clusters are high, but correlations between the comorbidity cluster and other clusters is low. Predictors are measured at three time points: * before cystectomy, ** at time of cystectomy, *** post-cystectomy.

Fig 5 shows the MI network adjacency matrix as a heat-map and the hierarchical clustering of variables in the dataset, where a four-cluster division is highlighted. Mean MI within the four clusters (purple, green, blue, red) is 0.624, 0.218, 0.0243, and 0.679, respectively, therefore the blue cluster is comprised of significantly less correlated variables than the other clusters. The purple cluster consists of pathologic staging variables which form the most correlated set of BCa-specific predictors (1-6 in Fig 5). BCa variables (purple, green and blue clusters) have an average MI of 0.0337 with each other, and an average MI of 0.00570 with the set of comorbidity variables (red cluster), marking the first division in the clustering. This suggests that patients’ preexisting comorbidities are not strongly related to BCa variables.

In contrast to comorbidity factors which are strongly associated with each other, the mean MI between predictors and the binary 5-year recurrence target variable (43 in Fig 5) is 0.01100, and 0.01863 between predictors and binary 5-year OS (44 in Fig 5), highlighting the difficulty in predicting long term BCa outcomes by using only preoperative and operative data. The association between 5-year recurrence and OS, MI = 0.257, is much higher than the mean MI between the predictors and either long term outcome. Both long-term outcomes are in the blue cluster (Fig 5) where there is a lack of strong associations among the variables aside from three sets of variables related to neoadjuvant chemotherapy (18-20 in Fig 5), adjuvant chemotherapy (29-31 in Fig 5), and radiation (21-23 in Fig 5) which are highly related because they are clinical re-classifications or sub-groupings of each other within the original variable’s domain. Aside from TNM 7th edition staging (7 in Fig 5) which is nearly identical to pT staging (TNM 5th edition, 8 in Fig 5), the highest associations with pT staging are pathologic stage subgroup (2 in Fig 5, MI = 0.236) and presence of pathologic carcinoma in situ (12 in Fig 5, MI = 0.1543). In contrast, clinical T stage (47 in Fig 5) does not have equally high MI with any of the other predictors except for the regrouped clinical staging variable (41 in Fig 5). The MI based heat-map and clustering in Fig 5 provides a system-wide view of the entire medical database for BCa patients and correlations between the predictors can be used to assess the quality of clinical measurement techniques.

### Correlations with long-term outcomes

**Fig 6:**
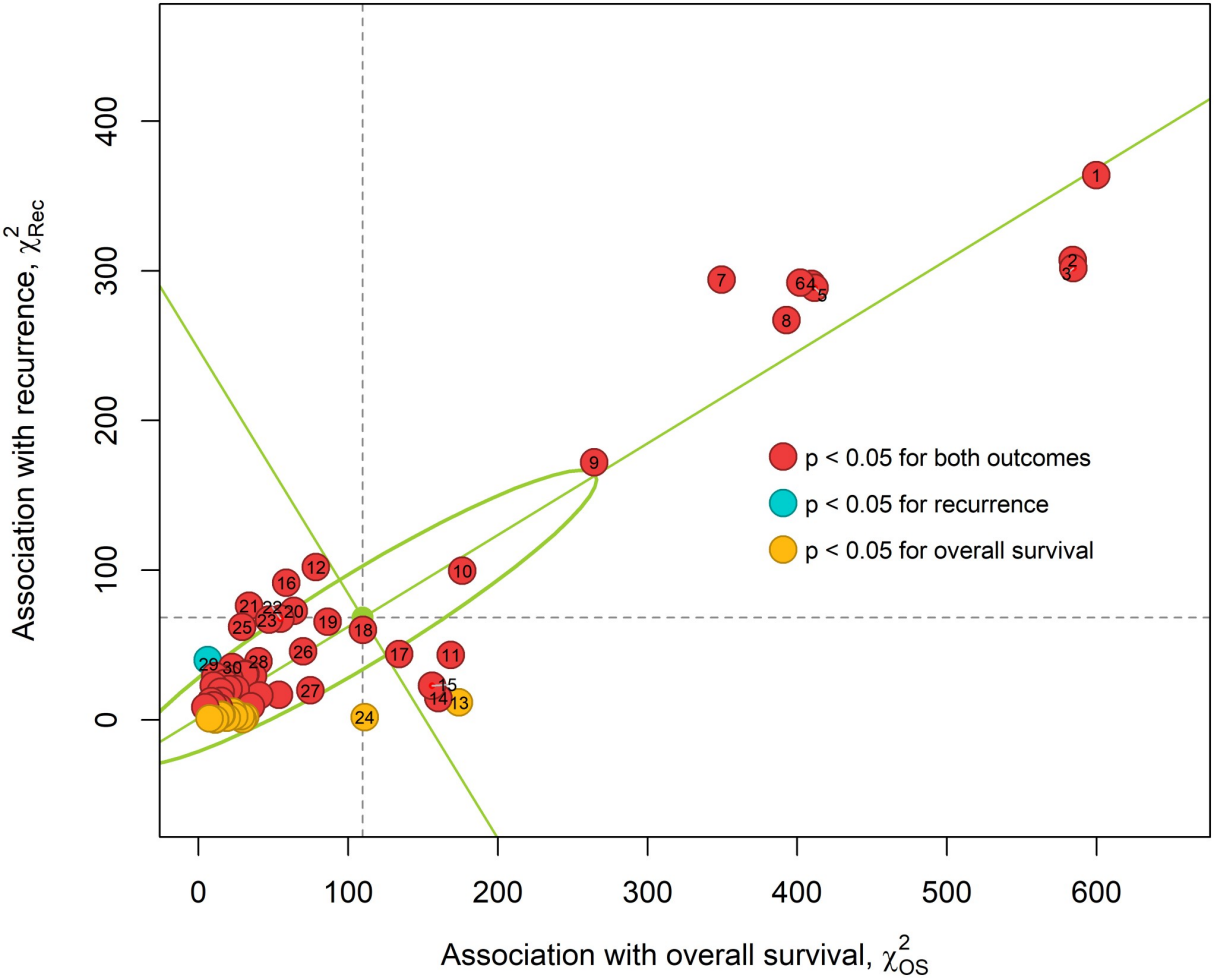
Association between predictors and the two long term binary 5-year outcomes, recurrence and OS as measured by the chi-squared test of independence. The predictors which have a statistical significance of p < 0.05 for both outcomes (45 predictors) are colored in red. Predictors of significance (p < 0.05) with recurrence (1 predictors) or OS (13 predictors) are shown in blue and yellow, respectively. The solid (green) lines are the two principal axes computed using singular value decomposition and the singular values are used to define the semi-major and semi-minor axes of the solid (green) ellipse. The horizontal and vertical dashed (gray) lines indicate the mean chi-squared for recurrence and OS respectively.

The chi-squared test of independence for binary 5-year recurrence (vertical axis) and binary 5-year OS (horizontal axis) in Fig 6 and Table 1 shows the relative importance of each predictor. The variance in chi-squared values is computed by singular value decomposition [18] and is shown by an ellipse whose axes are the standard deviations (SD) along the first (SD = 164.9) and second (SD = 26.8) principal components (green lines) in Fig 6A. Some predictors intrinsically contain more information about survival than recurrence, and vice versa. For instance, urinary diversion (rank 11) and age (rank 13) are more strongly correlated with OS than recurrence, nevertheless, they rank high due to large effect on survival.

**Table 1:** The ranked list of predictors by importance for 5-year outcomes.

Rank	Predictor	Recurrence		Overall survival	
		*χ*^2^	p-value	*χ*^2^	p-value
1	pathologic stage subgroup (OC, EV, N+) **	364.0	0.000	600.0	0.000
2	pT stage TNM 5th Edition **	307.3	0.000	584.0	0.000
3	pT stage TNM 7th Edition **	301.8	0.000	584.0	0.000
4	pN stage TNM 7th Edition **	291.3	0.000	410.0	0.000
5	pM stage TNM 7th Edition **	288.7	0.000	412.0	0.000
6	pN stage TNM 5th Edition **	292.0	0.000	402.0	0.000
7	# of positive lymph nodes **	294.1	0.000	349.0	0.000
8	pathologic positive lymph nodes **	267.0	0.000	393.0	0.000
9	pathologic lymphovascular invasion **	172.1	0.000	264.0	0.000
10	clinical T stage (preoperative) *	99.6	0.000	176.0	0.000
11	type of urinary diversion constructed **	43.4	0.000	169.0	0.000
12	neoadjuvant chemotherapy subgroups *	102.2	0.000	78.4	0.000
13	age at time of cystectomy (discretized) **	11.7	0.232	174.0	0.000
14	positive soft tissue surgical margin **	14.5	0.000	160.0	0.000
15	urinary diversion subgoups **	22.8	0.000	156.0	0.000
16	# of neoadjuvant chemo cycles *	91.6	0.000	58.6	0.000
17	type of surgical procedure **	44.0	0.000	134.0	0.000
18	clincial staging subgroup (preoperative) *	60.2	0.000	110.0	0.000
19	pathologic predominant cell type **	65.6	0.000	86.3	0.000
20	type of neoadjuvant chemotherapy *	72.8	0.000	63.7	0.000
21	adjuvant chemotherapy subgroups ***	76.3	0.000	33.8	0.000
22	type of adjuvant chemotherapy ***	67.6	0.000	54.7	0.000
23	pathologic carcinoma in situ **	66.9	0.000	47.1	0.000
24	pM stage TNM 5th Edition **	1.8	0.399	111.0	0.000
25	pathologic multifocal tumors **	62.1	0.000	29.2	0.000
26	clinical N Stage (preoperative) *	45.7	0.000	69.9	0.000
27	# lymph nodes removed **	19.7	0.006	74.7	0.000
28	intracorporeal diversion performed **	39.1	0.000	40.0	0.000
29	# of adjuvant chemo cycles ***	40.0	0.000	6.1	0.413
30	pathologic tumor grade **	35.2	0.000	22.5	0.000

Association between predictors and the two long term outcomes, recurrence and OS as measured by the chi-squared test of independence. Predictors are ranked by Eq 1. Predictors are measured at three time points:

* before cystectomy,

** at time of cystectomy,

*** post-cystectomy.

Pathologic stage subgroup (rank 1), which indicates whether patients have OC, EV, or N+ disease at time of cystectomy, has the largest $\chi_{Rec}^{2}$ and $\chi_{OS}^{2}$. Comparing pathologic stage (rank 2) to clinical staging (rank 10) reinforces the superiority of pathologic staging in differentiating patients by outcome as observed in the Kaplan-Meier curves (Figs 3A and 4A). The number of positive lymph nodes removed at time of cystectomy (rank 7) is significantly more correlated with recurrence than the total number of lymph nodes removed (rank 27). The predictors ranked 4-8 in Fig 6 have similar correlations with either outcome, and these predictors are part of the highly associated (purple) cluster in Fig 5. Appendix C in S1 File shows results of the same analysis for 1-year and 3-year binary outcomes, however most of the top correlates remain constant across these three time periods.

2757 patients who did not receive adjuvant chemotherapy had 5-year survival rate of 0.563 (95% CI [0.543, 0.583]), and 633 patients who did receive adjuvant chemotherapy had a 5-year survival rate of 0.450 (95% CI [0.411, 0.493]) (Fig E in S1 File). Since the prescription of adjuvant chemotherapy is limited to a homogeneous set of patients who are node positive at time of cystectomy, the association of adjuvant chemotherapy with recurrence and OS may be artificially high in this dataset.

Although the predictors with below average chi-squared values are less important than the others, they may still differentiate patients who are similar in the higher ranked variables. The lowest correlates of 5-year binary OS and recurrence are pathologic micropapillary ($\chi_{OS}^{2}$ = 4.52) and existence of diabetes ($\chi_{Rec}^{2}$ = 0.248) respectively. Overall the predictors have a weaker association with recurrence than OS. Since the MI between BCa specific predictors is small, even the predictors with small chi-squared values add new information about a patient. However, this new information may not necessarily inform OS and recurrence.

### Predicting post-cystectomy recurrence

We evaluate the performance of machine learning models to predict post-cystectomy disease recurrence using preoperative and operative data as well as the type and number of adjuvant therapy cycles administered (Table 2). Both univariate (logistic regression) and more complex multivariate models (meta-classifiers in Table 2) are used to predict 1-, 3-, and 5-year recurrence. Pathologic stage subgroup (rank 3 in Fig 6) and pT stage (rank 2 in Fig 6) are used to create the univariate models and these have lower precision and F1 scores than the meta-classifiers. Furthermore, the single predictor models tend to suffer from imbalance between sensitivity and specificity. In contrast, all recurrence meta-classifiers have sensitivities and specificities over 70%. F1 scores improve with year perhaps due to a more even number of positive and negative recurrence cases in the corresponding datasets.

**Table 2:** Performance of machine learning models for predicting recurrence and survival.

Post-cystectomy outcome	Model	Year	Test set performance metrics			
			Sensitivity	Specificity	Precision	F1
**Recurrence**	Meta-classifier	1	0.739	0.714	0.388	0.508
	pT stage TNM 5th Edition	1	0.761	0.653	0.349	0.478
	pathologic stage subgroup	1	0.826	0.593	0.332	0.473
	Meta-classifier	3	0.720	0.708	0.535	0.613
	pathologic stage subgroup	3	0.774	0.631	0.493	0.602
	pT stage TNM 5th Edition	3	0.670	0.694	0.503	0.574
	Meta-classifier	5	0.700	0.702	0.588	0.636
	pathologic stage subgroup	5	0.744	0.611	0.537	0.623
	pT stage TNM 5th Edition	5	0.619	0.698	0.553	0.583
**Survival**	Meta-classifier	1	0.741	0.770	0.473	0.577
	pT stage TNM 5th Edition	1	0.739	0.672	0.387	0.506
	pathologic stage subgroup	1	0.805	0.602	0.362	0.499
	Meta-classifier	3	0.722	0.788	0.700	0.711
	pathologic stage subgroup	3	0.762	0.691	0.628	0.688
	pT stage TNM 5th Edition	3	0.696	0.739	0.646	0.670
	Meta-classifier	5	0.741	0.768	0.780	0.760
	pathologic stage subgroup	5	0.730	0.717	0.742	0.735
	pT stage TNM 5th Edition	5	0.664	0.766	0.759	0.708

Single predictor (pT stage and pathologic stage subgroup classifiers) and multiple predictor (Meta-classifier) models for predicting 1-, 3-, 5-year recurrence and survival after cystectomy. The performance of all models for a given year is ranked per F1 scores (2*precision*recall/(precision+recall)) as well as mean sensitivity, specificity, and precision on test sets from a 10-fold cross validation.

### Predicting post-cystectomy survival

Like the recurrence predictions, the meta-classifiers outperform the univariate models in predicting survival (Table 2), however the disparity is greater as the meta-classifiers have considerably higher performance metrics for all year predictions. Additionally, and unlike the recurrence models, the survival meta-classifiers have comparable precision and probability of detection, except for the 1-year survival models. The combination of high precision and sensitivity leads to significantly higher F1 scores for the 3- and 5-year survival meta-classifiers.

## Discussion

Although recurrence and OS are highly associated, preoperative and operative measurements generally do not relate equally to recurrence and OS, and the two outcomes should be assessed separately. The primary predictors of long-term outcomes are pathologic stage and its subgrouping into localized or metastatic conditions. However, the machine learning pipeline developed here can leverage less powerful predictors to improve accuracy of long term predictions. The benefit of having low MI between variables means that each variable offers unique information however the drawback is that each patient needs to be described by many variables and thus the prediction task becomes a higher dimensional problem, for which lack of data can greatly limit predictions of long-term outcomes. Clinical T stage offers a lower resolution signal than the true pathologic T stage, and this loss of information can be particularly impactful in cases where there is an underestimation of disease severity prior to surgery [19].

The sensitivity and specificity of all the survival meta-classifiers, and the 1-year recurrence meta-classifiers are considerably higher than 70%. Recurrence meta-classifiers are less accurate, perhaps, because of undetected metastatic disease at the time of cystectomy. 1-year meta-classifier predictions for both outcomes offer a better combination of sensitivity and specificity than the 3- and 5-year meta-classifiers. However, the later year models may also be used in the clinical setting to differentiate lower- and higher-risk patients due to higher precision scores.

In current clinical practice, post-radical cystectomy prognostication in the individual patient is informed by the best-evidence found in the literature [10, 20] which reflect probable outcomes in cohorts not the individual, or the prognostic nomogram which only calculates a 5-year outcome [9]. To improve upon this, we employ machine-learning algorithms to construct novel, patient prognostication models for survival and recurrence. Presently the international bladder cancer nomogram has proven to be a validation of multivariate approaches in predicting long term outcomes in the clinical setting [9, 21], and the models developed here offer higher resolution predictions which can assist post-cystectomy treatment and screening decisions.

Despite several machine learning research efforts in predicting outcomes of cancer patients there is a low penetration of such models in clinical practice [8]. There are two specific hurdles before current models can be deployed in a clinical setting, first, because the performance reported here reflects the quality of data collected at one center and to ensure the generalizability of the models, data from other institutions should also be studied. Combining additional datasets, such as the international BCa dataset [9], may also improve the performance of the algorithms due to general sparsity and low frequency of certain combinations of predictors in the present data. Secondly, the recurrence and survival models use a total of 42-54 predictors, therefore the standardized collection of these parameters must be ensured before the machine learning models can be deployed successfully in a clinical setting. Once these issues are addressed, machine learning models such as the ones developed here can be trained using similar BCa radical cystectomy datasets to predict the recurrence and survival of an individual patient using the pre-, peri-, and post-operative predictors.

The accuracy of predicting cancer recurrence, which may depend on several evolutionary steps beyond cystectomy, can undoubtedly be improved by combining genomic and molecular data, and this would be fruitful direction to pursue. The quality of the dataset, coupled with machine learning models in the present work, offers a benchmark of the value of current preoperative and operative patient assessment standards with respect to forecasting long term outcomes during the most vulnerable 5-year timespan in BCa treatment post-cystectomy. Furthermore, due to the absence of widely recognized biomarkers for BCa [22], clinicopathological-based predictions of clinical outcomes as shown here set the standard for long-term personalized predictions in BCa. If deployed correctly, machine learning models can transform preoperative and operative data into accurate predictions and mitigate post-cystectomy burden of BCa.

## Supporting information

S1 File Supporting information file. (PDF)

## References

[1] Siegel RL, Miller KD, Fedewa SA, Ahnen DJ, Meester RG, Barzi A, et al Colorectal cancer statistics, 2017. CA: a cancer journal for clinicians. 2017;67(3):177–193. 28248415 10.3322/caac.21395

[2] Cancer Facts & Figures 2018. Atlanta: American Cancer Society; 2018.

[3] Dobruch J, Daneshmand S, Fisch M, Lotan Y, Noon AP, Resnick MJ, et al Gender and bladder cancer: a collaborative review of etiology, biology, and outcomes. European urology. 2016;69(2):300–310. 10.1016/j.eururo.2015.08.037 26346676

[4] Stenzl A, Cowan NC, De Santis M, Kuczyk MA, Merseburger AS, Ribal MJ, et al Treatment of muscle-invasive and metastatic bladder cancer: update of the EAU guidelines. European urology. 2011;59(6):1009–1018. 10.1016/j.eururo.2011.03.023 21454009

[5] Herr HW, Faulkner JR, Grossman HB, Natale RB, deVere White R, Sarosdy MF, et al Surgical factors influence bladder cancer outcomes: a cooperative group report. Journal of clinical oncology. 2004;22(14):2781–2789. 10.1200/JCO.2004.11.024 15199091

[6] Bader P, Burkhard FC, Markwalder R, Studer UE. Disease progression and survival of patients with positive lymph nodes after radical prostatectomy. Is there a chance of cure?. The Journal of urology. 2003;169(3):849–854. 12576797 10.1097/01.ju.0000049032.38743.c7

[7] Cruz JA, Wishart DS. Applications of machine learning in cancer prediction and prognosis. Cancer informatics. 2006;2:117693510600200030. 10.1177/117693510600200030 PMC2675494 19458758

[8] Kourou K, Exarchos TP, Exarchos KP, Karamouzis MV, Fotiadis DI. Machine learning applications in cancer prognosis and prediction. Computational and structural biotechnology journal. 2015;13:8–17. 10.1016/j.csbj.2014.11.005 25750696 PMC4348437

[9] Bochner BH, Kattan MW, Vora KC. Postoperative nomogram predicting risk of recurrence after radical cystectomy for bladder cancer. Journal of clinical oncology: official journal of the American Society of Clinical Oncology. 2006;24(24):3967–3972. 10.1200/JCO.2005.05.3884 16864855

[10] Stein JP, Lieskovsky G, Cote R, Groshen S, Feng AC, Boyd S, et al Radical cystectomy in the treatment of invasive bladder cancer: long-term results in 1,054 patients. Journal of clinical oncology. 2001;19(3):666–675. 10.1200/JCO.2001.19.3.666 11157016

[11] Butte AJ, Kohane IS. Mutual information relevance networks: functional genomic clustering using pairwise entropy measurements In: Biocomputing 2000. World Scientific; 1999 p. 418–429. 10.1142/9789814447331_0040 10902190

[12] Moriyama M, Hoshida Y, Otsuka M, Nishimura S, Kato N, Goto T, et al Relevance network between chemosensitivity and transcriptome in human hepatoma cells1. Molecular Cancer Therapeutics. 2003;2(2):199–205. 12589037

[13] Horvath S. Weighted network analysis: applications in genomics and systems biology. Springer Science & Business Media; 2011.

[14] Talukder AK, Agarwal M, Buetow KH, Denèfle PP. Tracking Cancer Genetic Evolution using OncoTrack. Scientific reports. 2016;6:srep29647. 10.1038/srep29647 PMC4944131 27412732

[15] Martin L, Gloor GB, Dunn S, Wahl LM. Using information theory to search for co-evolving residues in proteins. Bioinformatics. 2005;21(22):4116–4124. 10.1093/bioinformatics/bti671 16159918

[16] Pedregosa F, Varoquaux G, Gramfort A, Michel V, Thirion B, Grisel O, et al Scikit-learn: Machine learning in Python. Journal of machine learning research. 2011;12(Oct):2825–2830.

[17] Age Standards for Survival. 2018;.

[18] Akritas AG, Malaschonok GI. Applications of singular-value decomposition (SVD). Mathematics and computers in simulation. 2004;67(1-2):15–31. 10.1016/j.matcom.2004.05.005

[19] Svatek RS, Shariat SF, Novara G, Skinner EC, Fradet Y, Bastian PJ, et al Discrepancy between clinical and pathological stage: external validation of the impact on prognosis in an international radical cystectomy cohort. BJU international. 2011;107(6):898–904. 10.1111/j.1464-410X.2010.09628.x 21244604

[20] Quek ML, Stein JP, Nichols PW, Cai J, Miranda G, Groshen S, et al Prognostic significance of lymphovascular invasion of bladder cancer treated with radical cystectomy. The Journal of urology. 2005;174(1):103–106. 10.1097/01.ju.0000163267.93769.d8 15947587

[21] Vickers AJ, Cronin AM, Kattan MW, Gonen M, Scardino PT, Milowsky MI, et al Clinical benefits of a multivariate prediction model for bladder cancer: a decision analytic approach. Cancer. 2009;115(23):5460–5469. 10.1002/cncr.24615 19823979 PMC2785133

[22] Cheng L, Davison DD, Adams J, Lopez-Beltran A, Wang L, Montironi R, et al Biomarkers in bladder cancer: translational and clinical implications. Critical reviews in oncology/hematology. 2014;89(1):73–111. 10.1016/j.critrevonc.2013.08.008 24029603

