# Supplementary material for: Machine learning models for predicting post-cystectomy recurrence and survival in bladder cancer patients
Source: PLoS One. 2019 Feb 20;14(2):e0210976. doi: 10.1371/journal.pone.0210976 (PMC6382101; doi:10.1371/journal.pone.0210976)
Supplement: S1 File — (PDF) [file pone.0210976.s001.pdf]

Machine learning models for predicting post-cystectomy recurrence and survival in bladder cancer patients

Zaki Hasnain[1], Jeremy Mason[2], Karanvir Gill[2], Gus Miranda[3], Inderbir S. Gill[3], Peter Kuhn[1,2,3,4,5*], Paul K. Newton[1,5,6]

1 Department of Aerospace and Mechanical Engineering, Viterbi School of Engineering, University of Southern California, University Park Campus, Los Angeles, CA, USA
2 Department of Biological Sciences, Dornsife College of Letters, Arts, and Sciences, University of Southern California, University Park Campus, Los Angeles, CA, USA
3 USC Institute of Urology, Keck School of Medicine, University of Southern California, Health Sciences Campus, Los Angeles, CA, USA
4 Department of Biomedical Engineering, Viterbi School of Engineering, University of Southern California, University Park Campus, Los Angeles, CA, USA
5 Norris Comprehensive Cancer Center, Keck School of Medicine, University of Southern California, Health Sciences Campus, Los Angeles, CA, USA
6 Department of Mathematics, University of Southern California, University Park Campus, Los Angeles, CA, USA

* Corresponding author. pkuhn@usc.edu (PK)

Supporting Information File 1

Appendix A. Machine learning process for finding best model. The feature selection process for generating datasets for machine learning is described here. MI is calculated between p = 73 predictors and used to cluster variables into K = 60 clusters. MI is also calculated between p = 73 predictors and RecX (x = 1, 3, 5 years) and SurvX (x = 1, 3, 5 years). Correlated predictors are removed by selecting a single predictor from K = 60 clusters whose MI with the outcome is the highest. Irrelevant predictors are removed by pruning all predictors whose MI with the outcome is below a threshold (MI = 0.006 for RecX, MI = 0.003 for SurvX). These thresholds are selected heuristically, with some trial and error. The choice of number of clusters is another decision which can be explored with further trial and error computations, however this task was found to be too demanding. Therefore, it may be possible to achieve similar performance with more strict removal of correlated predictors by picking a smaller number of correlated clusters, K to prune from. All p = 73 predictors are categorical except for age, therefore discretized (10 bins with equal frequency) and continuous versions of age were tried separately.

Class-imbalance was overcome by random oversampling to achieve a ratio of minority to majority class k = 0.8, 0.9, or 1.0. Number of patients with recurrences (1) and no recurrences (0) in the 1-, 3-, and 5-year recurrence datasets are: (0: 2469, 1: 602) for 1-year, (0: 2020, 1: 935) for 3-year, (0: 1683, 1: 1012) for 5-year. Number of surviving (0) and not surviving (1) patients in the 1-, 3-, and 5-year survival datasets are: (0: 2503, 1: 698) for 1-year, (0: 1821, 1: 1245) for 3-year, (0: 1318, 1: 1462) for 5-year.

Thirteen base models include: SVM (polynomial, sigmoid, radial basis function kernels), KNN, AdaBoost, gradient boosted trees, random forest, pathologic stage based logistic regression, pathologic stage subgroup based logistic regression, and bagged versions of SVM and KNN. The main challenge in this study was to overcome the skewed performance of base models towards high sensitivity or high specificity. Consequently, a series of ensemble learning techniques are employed to take advantage of the base models. Nine different majority voting based mixture-of-expert (MOE) ensemble models, and 13 different logistic regression and SVM based stacking ensemble models are created using the results of the base models. Finally, the meta-classifiers are created by using a triplet of: a base model, a MOE ensemble model, and a stacking ensemble model. There are 1287 such triplets, and we find the performance of these models using nested k-fold cross validation, and the best models from this list are reported. These ensemble methods prove successful in overcoming the skewed base model performance. Due to the medial nature of the dataset, the predictors are categorical and the addition of numerical predictors directly from imaging and biopsy procedures may improve performance.

Table A. Dataset description.

	All	P0	Pa	PIS	P1	P2a	P2b	P3a	P3b	P4a	P4b
	(3503)	(334)	(94)	(404)	(481)	(300)	(389)	(357)	(536)	(451)	(141)
**Age (yr)**											
Median	68	66	67	67	66	67	67	70	69	70	68
Range	23-95	25-92	34-87	27-94	33-95	31-89	30-92	38-91	31-92	38-94	23-95
**Gender (n)**											
Female	757	66	16	62	91	52	99	97	171	70	30
Male	2746	268	78	342	390	248	290	260	365	381	111
**Histology (n)**											
Adeno	40	3	1	0	4	1	6	7	7	7	4
Neuro	42	7	0	0	0	3	5	5	12	10	0
Sarcoma	19	2	0	0	1	5	3	3	1	3	1
Squamous	90	3	0	2	5	5	16	7	27	13	11
Urothelial	3301	315	92	402	470	286	358	335	486	417	125
Other	11	4	1	0	1	0	1	0	3	1	0
**pN stage (n)**											
Nx	151	14	5	11	16	8	8	5	16	30	32
N0	2477	309	88	381	435	248	293	206	284	181	49
N1	220	3	0	5	14	18	29	41	59	37	11
N2	639	7	1	7	16	25	59	101	176	197	46
N3	16	1	0	0	0	1	0	4	1	6	3
**Pathologic subgroup (n)**											
OC	1812	323	93	391	450	254	299	0	0	0	0
EV	760	0	0	0	0	0	0	208	293	201	58
N+	931	11	1	13	31	46	90	149	243	250	83
**Radiation (n)**											
Neoadjuvant	325	35	7	34	50	26	33	20	35	54	28
Adjuvant	54	0	0	0	2	2	1	5	14	8	20
**Chemotherapy (n)**											
Neoadjuvant	519	93	7	59	32	32	39	52	62	89	52
Adjuvant	633	8	3	12	26	37	80	106	178	142	36
**Smoker (n)**											
Current	674	52	19	61	88	71	86	61	127	74	35
Never	807	84	18	82	117	57	92	94	132	94	34
Previous	1891	188	55	249	255	157	195	189	264	267	63
**Metastasis (n)**											
Total Patients	1061	36	16	67	96	68	100	154	242	231	48
Bone	332	6	1	14	27	18	26	54	91	80	14
Pelvis	308	8	1	15	13	14	33	48	83	70	23
Lung	288	11	4	19	29	24	29	43	70	49	10
Liver	255	12	2	20	24	8	19	35	68	56	10
Retro	208	6	0	21	19	12	14	42	44	43	7
LN (reg)	134	3	2	9	16	7	9	23	27	30	7
LN (dist)	95	4	0	5	5	12	9	19	14	22	5
Peritoneum	93	7	0	6	2	5	8	10	18	28	9
Urethra	88	5	7	15	20	11	5	2	8	14	1
Brain	65	1	0	4	6	2	10	11	14	14	3
UT	61	3	5	11	12	11	2	2	8	7	0
Adrenal	34	0	0	3	2	2	2	6	10	6	3
Other	174	7	2	11	15	14	13	29	37	36	10

Patient and tumor characteristics by pathologic T stage.

Appendix B Clinical vs Pathologic staging trend since 1970s. Clinical staging (staging based on a combination of imaging and transurethral resection) and pathological staging (staging done at time of cystectomy) do not always classify patients in the same stages, and clinical staging may underestimate (shown in blue), agree (shown in green) or overestimate (shown in red). Clinical T stages are: T0, Ta, TIS, T1, T2a, T2b, T3a, T3b, T4a, T4b. Pathologic stages are: P0, Pa, PIS, P1, P2a, P2b, P3a, P3b, P4a, P4b. TX and PX indicate patients whose stage was declared unknown, and these patients are removed for this analysis resulting in a dataset of N = 3417 patients for Fig. A and all concordance analyses between clinical and pathologic staging.

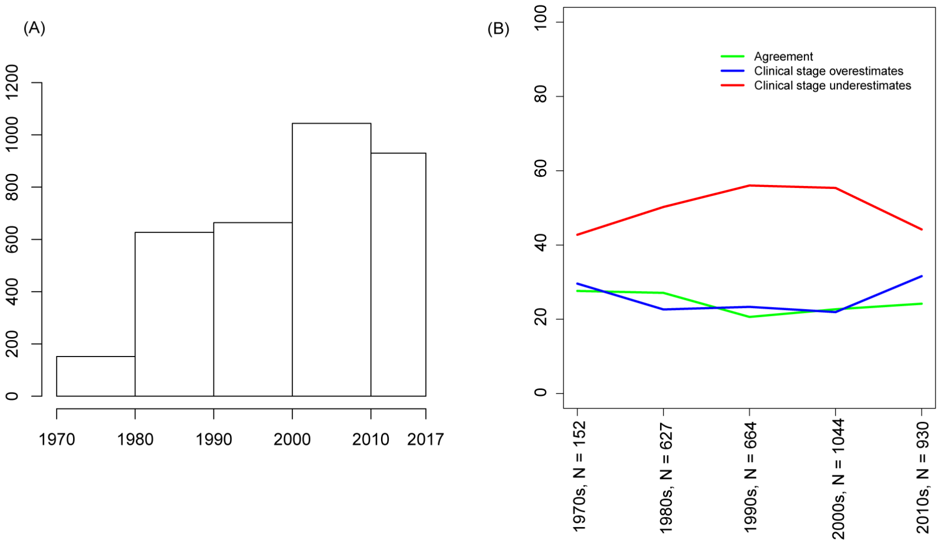

Fig A. Discrepancy in staging over time. (A) Histogram of number of patients undergoing cystectomy for each decade of data collection. (B) The agreement between clinical and pathological staging over the period of data collection (1971-2016).

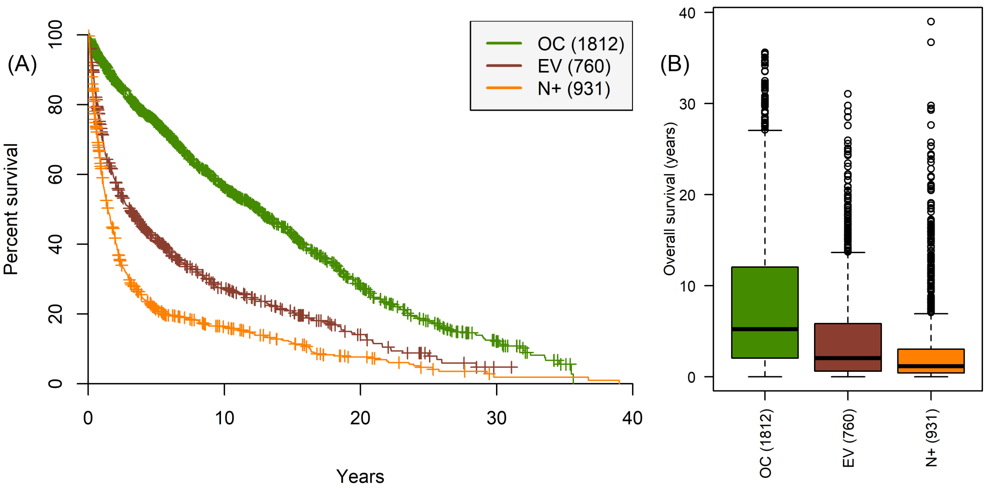

at risk (# censored)

OC	1812 (3)	564 (648)	160 (839)	30 (897)	0 (915)
EV	760 (0)	111 (180)	28 (223	1 (236)	0 (237)
N+	931 (1)	86 (152)	22 (179)	2 (188)	0 (188)

Fig B. Bladder stage survival analysis. (A) Kaplan-Meier survival computed by bladder stage at time of cystectomy subgrouped into OC (T0, Ta, TIS, T1, T2a, T2b), EV (T3a, T3b, T4a, T4b), N+ irrespective of stage (metastasized) (p-value < 0.001). (B) Boxplot of OS by bladder stage at time of cystectomy.

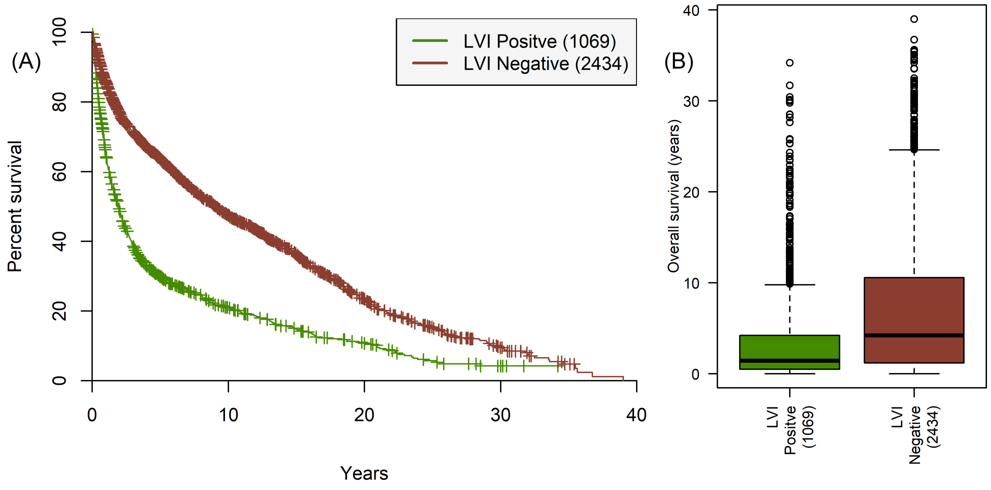

at risk (# censored)

LVI Positive	1069 (1)	116 (218)	38 (249)	4 (265)	0 (269)
LVI Negative	2434 (3)	645 (762)	172 (992)	29 (1056)	0 (1071)

Fig C. Lymphovascular invasion survival analysis. (A) Kaplan-Meier survival computed by lymphovascular invasion. (B) Boxplot of OS by lymphovascular invasion.

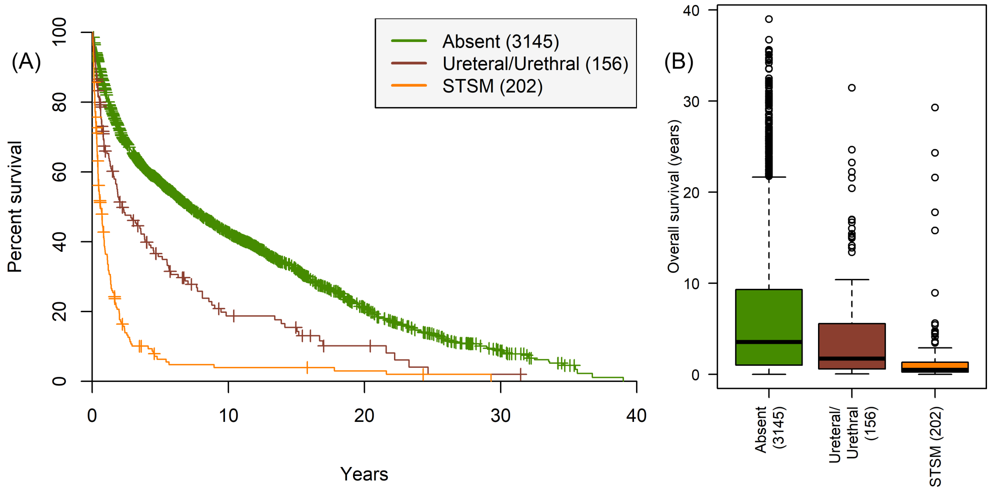

at risk (# censored)

Absent	3145 (4)	738 (927)	201 (1182)	32 (1260)	0 (1278)
Ureteral/ Urethral	156 (0)	18 (29)	6 (34)	1 (35)	0 (36)
STSM	202 (0)	5 (24)	3 (25)	0 (26)	0 (26)

Fig D. Surgical margin survival analysis (A) Kaplan-Meier survival computed by soft tissue surgical margin. (B) Boxplot of OS by soft tissue surgical margin.

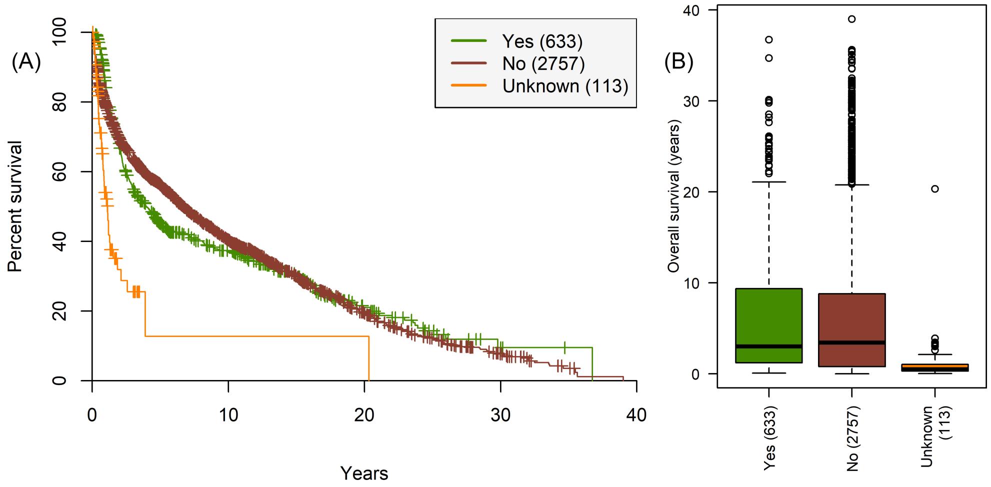

at risk (# censored)

Yes	633 (0)	153 (123)	40 (187)	3 (211)	0 (213)
No	2757 (3)	607 (795)	169 (992)	30 (1048)	0 (1065)
Unknown	113 (1)	1 (62)	1 (62)	0 (62)	0 (62)

Fig E. Adjuvant chemotherapy survival analysis. (A) Kaplan-Meier survival computed by receipt of adjuvant chemotherapy. (B) Boxplot of OS by receipt of adjuvant chemotherapy.

Appendix C. Chi-squared test of independence: predictors vs. recurrence and overall survival. Complete results of the Chi-squared test of independence between predictors and binary recurrence and binary OS are shown here. Top 30 correlated predictors for 1-year and 3-year outcomes are shown in Table B and Table C respectively, these can be compared to correlations for 5-year outcomes shown in Table 1 in the main text. The complete list of 59 predictors for correlations with 5-year binary outcomes are shown in Table D and Table E. Note that the continuous predictor age is discretized into 10 bins of equal frequency and predictors are ranked by (Eqs.1, 2, 3). Patients who left the study before 1, 3, and 5 years, had no recurrence, and were alive at that point were omitted from the datasets used to calculate correlation with recurrence - resulting in 3071, 2955, and 2695 patients in the recurrence calculations for 1-, 3-, and 5-year correlations. Similarly, patients who left the study before 1, 3, and 5 years, and were alive at that point were omitted from the datasets used to calculate correlation with OS - resulting in 3201, 3066, and 2780 patients in the OS calculations for 1-, 3-, and 5-year correlations. Furthermore, The tests for association with OS exclude the 4 patients who are missing survival data, and the tests for recurrence exclude 163 patients whose recurrence status is unknown.

Table B. The ranked list of predictors by importance for 1-year outcomes.

		Recurrence		Overall survival	
Rank	Predictor	χ^2	p-value	χ^2	p-value
1	pT stage TNM 5th Edition **	370.1	0.000	558.0	0.000
2	pT stage TNM 7th Edition **	363.1	0.000	564.0	0.000
3	pathologic stage subgroup (OC, EV, N+) **	368.2	0.000	392.0	0.000
4	pM stage TNM 7th Edition **	259.6	0.000	301.0	0.000
5	pN stage TNM 7th Edition **	261.3	0.000	297.0	0.000
6	pN stage TNM 5th Edition **	265.4	0.000	289.0	0.000
7	# of positive lymph nodes **	268.0	0.000	230.0	0.000
8	pathologic positive lymph nodes **	247.6	0.000	259.0	0.000
9	pathologic lymphovascular invasion **	168.2	0.000	185.0	0.000
10	pM stage TNM 5th Edition **	34.7	0.000	251.0	0.000
11	clinical T stage (preoperative) *	138.1	0.000	182.0	0.000
12	positive soft tissue surgical margin **	48.6	0.000	246.0	0.000
13	clincial staging subgroup (preoperative) *	109.2	0.000	142.0	0.000
14	urinary diversion subgoups **	39.8	0.000	163.0	0.000
15	clinical M stage (preoperative) *	63.6	0.000	121.0	0.000
16	# lymph nodes removed **	5.4	0.607	134.0	0.000
17	neoadjuvant chemotherapy subgroups *	87.2	0.000	58.5	0.000
18	type of surgical procedure **	26.4	0.002	120.0	0.000
19	clinical N Stage (preoperative) *	79.6	0.000	70.3	0.000
20	type of neoadjuvant chemotherapy *	80.0	0.000	45.0	0.000
21	# of neoadjuvant chemo cycles *	80.7	0.000	42.6	0.000
22	age at time of cystectomy (discretized) **	12.6	0.184	101.0	0.000
23	pathologic predominant cell type **	60.6	0.000	63.4	0.000
24	pathologic carcinoma in situ **	55.0	0.000	32.8	0.000
25	pathologic multifocal tumors **	51.6	0.000	12.0	0.002
26	type of adjuvant chemotherapy ***	10.4	0.005	58.9	0.000
27	type of urinary diversion constructed **	17.1	0.000	54.8	0.000
28	pathologic squamous differentiation **	35.4	0.000	36.5	0.000
29	# of adjuvant chemo cycles ***	6.4	0.375	55.5	0.000
30	indication for surgery **	25.4	0.000	27.7	0.000

Association between predictors and the two long term outcomes, recurrence and OS as measured by the chi-squared test of independence. Predictors are ranked by Eq.1. Predictors are measured at three time points: * before cystectomy, ** at time of cystectomy, *** post-cystectomy.

Table C. The ranked list of predictors by importance for 3-year outcomes.

Rank	Predictor	Recurrence χ^2	Recurrence p-value	Overall survival χ^2	Overall survival p-value
1	pathologic stage subgroup (OC, EV, N+) **	479.6	0.000	672.0	0.000
2	pT stage TNM 5th Edition **	405.2	0.000	663.0	0.000
3	pT stage TNM 7th Edition **	398.8	0.000	667.0	0.000
4	pN stage TNM 7th Edition **	379.2	0.000	488.0	0.000
5	pM stage TNM 7th Edition **	377.0	0.000	491.0	0.000
6	pN stage TNM 5th Edition **	380.0	0.000	480.0	0.000
7	pathologic positive lymph nodes **	355.0	0.000	465.0	0.000
8	# of positive lymph nodes **	380.3	0.000	415.0	0.000
9	pathologic lymphovascular invasion **	220.4	0.000	296.0	0.000
10	clinical T stage (preoperative) *	128.2	0.000	230.0	0.000
11	positive soft tissue surgical margin **	27.5	0.000	225.0	0.000
12	urinary diversion subgoups **	31.6	0.000	187.0	0.000
13	clincial staging subgroup (preoperative) *	75.5	0.000	151.0	0.000
14	pM stage TNM 5th Edition **	7.8	0.021	169.0	0.000
15	pathologic predominant cell type **	83.0	0.000	106.0	0.000
16	adjuvant chemotherapy subgroups ***	95.3	0.000	27.5	0.000
17	type of surgical procedure **	27.4	0.001	131.0	0.000
18	type of adjuvant chemotherapy ***	84.9	0.000	60.0	0.000
19	age at time of cystectomy (discretized) **	7.0	0.638	132.0	0.000
20	clinical N Stage (preoperative) *	59.3	0.000	92.6	0.000
21	# lymph nodes removed **	8.4	0.300	119.0	0.000
22	pathologic carcinoma in situ **	75.7	0.000	49.8	0.000
23	neoadjuvant chemotherapy subgroups *	70.6	0.000	52.1	0.000
24	# of neoadjuvant chemo cycles *	71.8	0.000	46.1	0.000
25	pathologic multifocal tumors **	71.3	0.000	31.6	0.000
26	type of neoadjuvant chemotherapy *	62.0	0.000	49.1	0.000
27	pathologic size of bladder tumor, dim-3 (cm) **	47.7	0.000	37.3	0.000
28	type of urinary diversion constructed **	15.9	0.000	72.8	0.000
29	clinical M stage (preoperative) *	27.0	0.000	63.3	0.000
30	pathologic size of bladder tumor, dim-2 (cm) **	45.4	0.000	34.6	0.000

Association between predictors and the two long term outcomes, recurrence and OS as measured by the chi-squared test of independence. Predictors are ranked by Eq.1. Predictors are measured at three time points: * before cystectomy, ** at time of cystectomy, *** post-cystectomy.

Table D. The ranked list of predictors by importance for 5-year outcomes. Ranked by Eq.1-3. Predictors 1-40.

		Recurrence			Overall survival		
Rank	Predictor	χ^2	p-value	V	χ^2	p-value	V
1	pathologic stage subgroup (OC, EV, N+) **	364.0	0.000	0.368	600.0	0.000	0.465
2	pT stage TNM 5th Edition **	307.3	0.000	0.338	584.0	0.000	0.458
3	pT stage TNM 7th Edition **	301.8	0.000	0.335	584.0	0.000	0.459
4	pN stage TNM 7th Edition **	291.3	0.000	0.329	410.0	0.000	0.384
5	pM stage TNM 7th Edition **	288.7	0.000	0.327	412.0	0.000	0.385
6	pN stage TNM 5th Edition **	292.0	0.000	0.329	402.0	0.000	0.380
7	# of positive lymph nodes **	294.1	0.000	0.330	349.0	0.000	0.355
8	pathologic positive lymph nodes **	267.0	0.000	0.315	393.0	0.000	0.376
9	pathologic lymphovascular invasion **	172.1	0.000	0.253	264.0	0.000	0.308
10	clinical T stage (preoperative) *	99.6	0.000	0.192	176.0	0.000	0.252
11	type of urinary diversion constructed **	43.4	0.000	0.127	169.0	0.000	0.246
12	neoadjuvant chemotherapy subgroups *	102.2	0.000	0.195	78.4	0.000	0.168
13	age at time of cystectomy (discretized) **	11.7	0.232	0.066	174.0	0.000	0.250
14	positive soft tissue surgical margin **	14.5	0.000	0.073	160.0	0.000	0.240
15	urinary diversion subgoups **	22.8	0.000	0.092	156.0	0.000	0.237
16	# of neoadjuvant chemo cycles *	91.6	0.000	0.184	58.6	0.000	0.145
17	type of surgical procedure **	44.0	0.000	0.128	134.0	0.000	0.220
18	clincial staging subgroup (preoperative) *	60.2	0.000	0.149	110.0	0.000	0.199
19	pathologic predominant cell type **	65.6	0.000	0.156	86.3	0.000	0.176
20	type of neoadjuvant chemotherapy *	72.8	0.000	0.164	63.7	0.000	0.151
21	adjuvant chemotherapy subgroups ***	76.3	0.000	0.168	33.8	0.000	0.110
22	type of adjuvant chemotherapy ***	67.6	0.000	0.158	54.7	0.000	0.140
23	pathologic carcinoma in situ **	66.9	0.000	0.158	47.1	0.000	0.130
24	pM stage TNM 5th Edition **	1.8	0.399	0.026	111.0	0.000	0.200
25	pathologic multifocal tumors **	62.1	0.000	0.152	29.2	0.000	0.102
26	clinical N Stage (preoperative) *	45.7	0.000	0.130	69.9	0.000	0.159
27	# lymph nodes removed **	19.7	0.006	0.085	74.7	0.000	0.164
28	intracorporeal diversion performed **	39.1	0.000	0.120	40.0	0.000	0.120
29	# of adjuvant chemo cycles ***	40.0	0.000	0.122	6.1	0.413	0.047
30	pathologic tumor grade **	35.2	0.000	0.114	22.5	0.000	0.090
31	current urinary diversion performed **	16.8	0.000	0.079	53.8	0.000	0.139
32	robotic surgical procedure **	29.3	0.000	0.104	36.4	0.000	0.114
33	pathologic size of bladder tumor, dim-3 (cm) **	30.6	0.000	0.107	31.3	0.000	0.106
34	pathologic size of bladder tumor, dim-2 (cm) **	30.3	0.000	0.106	30.9	0.000	0.105
35	clinical M stage (preoperative) *	16.3	0.000	0.078	40.6	0.000	0.121
36	predominant clinical cell type (preoperative) *	27.4	0.000	0.101	20.6	0.004	0.086
37	pathologic glandular differentiation **	29.3	0.000	0.104	11.1	0.011	0.063
38	pathologic squamous differentiation **	24.5	0.000	0.095	17.6	0.000	0.080
39	indication for surgery **	20.6	0.000	0.087	24.9	0.000	0.095
40	pathologic other variant histology **	20.6	0.004	0.087	20.0	0.006	0.085

Predictors are measured at three time points: * before cystectomy, ** at time of cystectomy, *** post-cystectomy.

Table E. The ranked list of predictors by importance for 5-year outcomes. Ranked by Eq.1-3. Predictors 41-59.

Rank	Predictor	Recurrence			Overall survival		
		χ^2	p-value	V	χ^2	p-value	V
41	clinical CIS *	9.1	0.003	0.058	35.1	0.000	0.112
42	pathologic neuroendocrine differentiation **	22.9	0.000	0.092	10.3	0.006	0.061
43	pathologic size of bladder tumor, dim-1 (cm) **	18.7	0.002	0.083	14.8	0.011	0.073
44	Charlson: renal disease, moderate or severe *	2.0	0.361	0.027	31.2	0.000	0.106
45	Charlson: any other malignancy *	0.4	0.831	0.012	29.5	0.000	0.103
46	any prior pelvic radiation *	2.5	0.116	0.030	28.3	0.000	0.101
47	history of smoking *	12.7	0.005	0.069	15.0	0.002	0.073
48	Charlson: chronic obstructive pulmonary disease *	5.5	0.064	0.045	22.8	0.000	0.091
49	# of neoadjuvant radiation to bladder *	2.4	0.795	0.030	23.9	0.000	0.093
50	gender **	12.5	0.000	0.068	8.4	0.004	0.055
51	adjuvant radiation post cystectomy ***	1.4	0.242	0.023	19.0	0.000	0.083
52	Charlson: connective tissue disorder *	8.3	0.016	0.056	13.6	0.001	0.070
53	pathologic # of tumors **	9.7	0.046	0.060	10.0	0.041	0.060
54	Charlson: congestive heart failure *	3.5	0.174	0.036	15.4	0.000	0.074
55	Charlson: myocardial infarction *	3.3	0.194	0.035	14.8	0.000	0.073
56	pathologic micropapillary **	8.5	0.003	0.056	4.5	0.034	0.040
57	Charlson: diabetes *	0.2	0.884	0.010	11.3	0.003	0.064
58	Charlson: peripheral vascular disease *	0.3	0.845	0.011	11.0	0.004	0.063
59	neoadjuvant bladder radiation *	1.0	0.319	0.019	7.4	0.006	0.052

Predictors are measured at three time points: * before cystectomy, ** at time of cystectomy, *** post-cystectomy.

Included here is a ranking of the predictors based on Cramer's V shown in Appendix C and listed in Table F and Table G. Cramer's V normalizes the chi-squared values by the number of categories in the predictors and offers an alternate ranking of the predictor importance with recurrence and overall survival. A composite predictor ranking,

$$rank_i = \sqrt{(\bar{V}_{Rec,i}^2)^2 + (\bar{V}_{OS,i}^2)^2} \tag{1}$$

based on Cramer's V values for recurrence (V_{Rec}),

$$\bar{V}_{Rec,i} = \frac{V_{Rec,i}}{\sigma_{V_{\text{Rec}}}} \tag{2}$$

and the Cramer's V values for overall survival (V_{OS}),

$$\bar{V}_{OS,i} = \frac{V_{OS,i}}{\sigma_{V_{\text{OS}}}} \tag{3}$$

is used to identify predictor importance. The Cramer's V values for both outcomes are normalized by their respective standard deviations (Eqs.1, 2, 3) to weigh the effect of both outcomes equally.

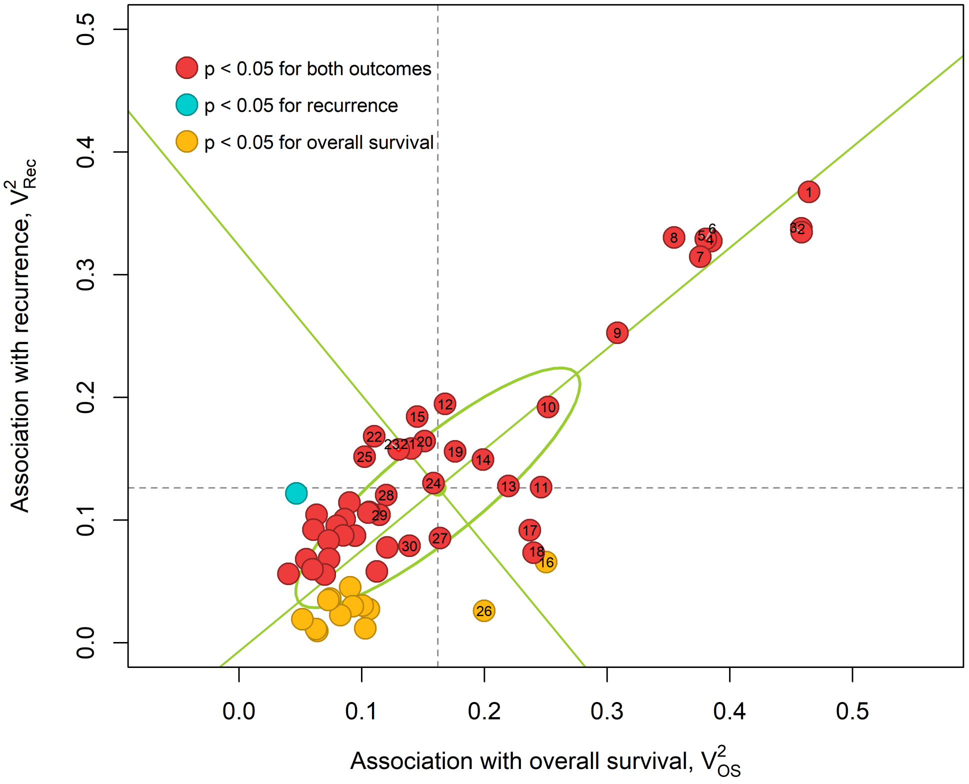

Fig F. Predictors ranked by Cramer's V.

Table F. The ranked list of predictors by importance for 5-year outcomes. Ranked by Eq.4-6. Predictors 1-40.

		Recurrence			Overall survival		
Rank	Predictor	χ^2	p-value	V	χ^2	p-value	V
1	pathologic stage subgroup (OC, EV, N+) **	364.0	0.000	0.368	600.0	0.000	0.465
2	pT stage TNM 5th Edition **	307.3	0.000	0.338	584.0	0.000	0.458
3	pT stage TNM 7th Edition **	301.8	0.000	0.335	584.0	0.000	0.459
4	pN stage TNM 7th Edition **	291.3	0.000	0.329	410.0	0.000	0.384
5	pM stage TNM 7th Edition **	288.7	0.000	0.327	412.0	0.000	0.385
6	pN stage TNM 5th Edition **	292.0	0.000	0.329	402.0	0.000	0.380
7	pathologic positive lymph nodes **	267.0	0.000	0.315	393.0	0.000	0.376
8	# of positive lymph nodes **	294.1	0.000	0.330	349.0	0.000	0.355
9	pathologic lymphovascular invasion **	172.1	0.000	0.253	264.0	0.000	0.308
10	clinical T stage (preoperative) *	99.6	0.000	0.192	176.0	0.000	0.252
11	type of urinary diversion constructed **	43.4	0.000	0.127	169.0	0.000	0.246
12	neoadjuvant chemotherapy subgroups *	102.2	0.000	0.195	78.4	0.000	0.168
13	type of surgical procedure **	44.0	0.000	0.128	134.0	0.000	0.220
14	clincial staging subgroup (preoperative) *	60.2	0.000	0.149	110.0	0.000	0.199
15	# of neoadjuvant chemo cycles *	91.6	0.000	0.184	58.6	0.000	0.145
16	age at time of cystectomy (discretized) **	11.7	0.232	0.066	174.0	0.000	0.250
17	urinary diversion subgoups **	22.8	0.000	0.092	156.0	0.000	0.237
18	positive soft tissue surgical margin **	14.5	0.000	0.073	160.0	0.000	0.240
19	pathologic predominant cell type **	65.6	0.000	0.156	86.3	0.000	0.176
20	type of neoadjuvant chemotherapy *	72.8	0.000	0.164	63.7	0.000	0.151
21	type of adjuvant chemotherapy ***	67.6	0.000	0.158	54.7	0.000	0.140
22	adjuvant chemotherapy subgroups ***	76.3	0.000	0.168	33.8	0.000	0.110
23	pathologic carcinoma in situ **	66.9	0.000	0.158	47.1	0.000	0.130
24	clinical N Stage (preoperative) *	45.7	0.000	0.130	69.9	0.000	0.159
25	pathologic multifocal tumors **	62.1	0.000	0.152	29.2	0.000	0.102
26	pM stage TNM 5th Edition **	1.8	0.399	0.026	111.0	0.000	0.200
27	# lymph nodes removed **	19.7	0.006	0.085	74.7	0.000	0.164
28	intracorporeal diversion performed **	39.1	0.000	0.120	40.0	0.000	0.120
29	robotic surgical procedure **	29.3	0.000	0.104	36.4	0.000	0.114
30	current urinary diversion performed **	16.8	0.000	0.079	53.8	0.000	0.139
31	pathologic size of bladder tumor, dim-3 (cm) **	30.6	0.000	0.107	31.3	0.000	0.106
32	pathologic size of bladder tumor, dim-2 (cm) **	30.3	0.000	0.106	30.9	0.000	0.105
33	pathologic tumor grade **	35.2	0.000	0.114	22.5	0.000	0.090
34	clinical M stage (preoperative) *	16.3	0.000	0.078	40.6	0.000	0.121
35	# of adjuvant chemo cycles ***	40.0	0.000	0.122	6.1	0.413	0.047
36	predominant clinical cell type (preoperative) *	27.4	0.000	0.101	20.6	0.004	0.086
37	indication for surgery **	20.6	0.000	0.087	24.9	0.000	0.095
38	pathologic glandular differentiation **	29.3	0.000	0.104	11.1	0.011	0.063
39	pathologic squamous differentiation **	24.5	0.000	0.095	17.6	0.000	0.080
40	pathologic other variant histology **	20.6	0.004	0.087	20.0	0.006	0.085

Predictors are measured at three time points: * before cystectomy, ** at time of cystectomy, *** post-cystectomy.

Table G. The ranked list of predictors by importance for 5-year outcomes. Ranked by Eq.4-6. Predictors 41-59.

		**Recurrence**			**Overall survival**		
Rank	Predictor	χ^2	p-value	V	χ^2	p-value	V
41	clinical CIS *	9.1	0.003	0.058	35.1	0.000	0.112
42	pathologic neuroendocrine differentiation **	22.9	0.000	0.092	10.3	0.006	0.061
43	pathologic size of bladder tumor, dim-1 (cm) **	18.7	0.002	0.083	14.8	0.011	0.073
44	Charlson: renal disease, moderate or severe *	2.0	0.361	0.027	31.2	0.000	0.106
45	history of smoking *	12.7	0.005	0.069	15.0	0.002	0.073
46	any prior pelvic radiation *	2.5	0.116	0.030	28.3	0.000	0.101
47	Charlson: chronic obstructive pulmonary disease *	5.5	0.064	0.045	22.8	0.000	0.091
48	Charlson: any other malignancy *	0.4	0.831	0.012	29.5	0.000	0.103
49	# of neoadjuvant radiation to bladder *	2.4	0.795	0.030	23.9	0.000	0.093
50	gender **	12.5	0.000	0.068	8.4	0.004	0.055
51	Charlson: connective tissue disorder *	8.3	0.016	0.056	13.6	0.001	0.070
52	pathologic # of tumors **	9.7	0.046	0.060	10.0	0.041	0.060
53	adjuvant radiation post cystectomy ***	1.4	0.242	0.023	19.0	0.000	0.083
54	Charlson: congestive heart failure *	3.5	0.174	0.036	15.4	0.000	0.074
55	Charlson: myocardial infarction *	3.3	0.194	0.035	14.8	0.000	0.073
56	pathologic micropapillary **	8.5	0.003	0.056	4.5	0.034	0.040
57	Charlson: diabetes *	0.2	0.884	0.010	11.3	0.003	0.064
58	Charlson: peripheral vascular disease *	0.3	0.845	0.011	11.0	0.004	0.063
59	neoadjuvant bladder radiation *	1.0	0.319	0.019	7.4	0.006	0.052

Predictors are measured at three time points: * before cystectomy, ** at time of cystectomy, *** post-cystectomy.

Table H. p-value of paired log rank tests for clinical T stage subgroups.

	Ta	TIS	T1	T2a	T2b	T3a	T3b	T4a	T4b
T0	0.656	0.896	0.442	0.049	4.00E-05	0.086	3.48E-05	3.85E-05	1.06E-06
Ta	———	0.505	0.732	0.030	1.12E-07	0.104	1.03E-07	3.10E-07	9.80E-08
TIS	———	———	0.152	6.29E-04	4.27E-12	0.029	2.48E-13	1.58E-11	6.78E-10
T1	———	———	———	2.03E-04	1.44E-15	0.078	0	9.44E-15	4.98E-10
T2a	———	———	———	———	8.71E-09	0.536	3.97E-09	5.56E-08	3.98E-06
T2b	———	———	———	———	———	0.232	0.519	0.699	0.024
T3a	———	———	———	———	———	———	0.199	0.207	0.018
T3b	———	———	———	———	———	———	———	0.928	0.055
T4a	———	———	———	———	———	———	———	———	0.061

Table I. p-value of paired log rank tests for pathologic T stage subgroups.

	Pa	PIS	P1	P2a	P2b	P3a	P3b	P4a	P4b
P0	0·638	0·666	0·047	0·027	2·47E-07	0	0	0	0
Pa	———	0·791	0·357	0·243	0·005	3·45E-09	3·29E-10	0	0
PIS	———	———	0·083	0·062	1·07E-07	0	0	0	0
P1	———	———	———	0·724	1·48E-04	0	0	0	0
P2a	———	———	———	———	0·004	1·13E-14	0	0	0
P2b	———	———	———	———	———	6·59E-09	5·18E-12	0	0
P3a	———	———	———	———	———	———	0·239	3·13E-07	0
P3b	———	———	———	———	———	———	———	8·12E-06	0
P4a	———	———	———	———	———	———	———	———	2·89E-13
